# Environmental reservoirs of pathogenic mycobacteria across the Ethiopian biogeographical landscape

**DOI:** 10.1371/journal.pone.0173811

**Published:** 2017-03-23

**Authors:** Hayley C. King, Tanya Khera-Butler, Phillip James, Brian B. Oakley, Girume Erenso, Abraham Aseffa, Rob Knight, Elizabeth M. Wellington, Orin Courtenay

**Affiliations:** 1 School of Life Sciences, University of Warwick, Coventry, United Kingdom; 2 College of Veterinary Medicine, Western University of Health Sciences, Pomona, California, United States of America; 3 St. Paul’s Hospital Millennium Medical College, Department of Microbiology, Immunology and Parasitology, Addis Ababa, Ethiopia; 4 Armauer Hansen Research Institute (AHRI), ALERT Campus, Addis Ababa, Ethiopia; 5 Department of Pediatrics and Department of Computer Science, University of California, San Diego, California, United States of America; University of Minnesota, UNITED STATES

## Abstract

The *Mycobacterium* genus comprises over one-hundred-and-fifty recognised species, the majority of which reside in the environment and many of which can be pathogenic to mammals. Some species of environmental mycobacteria may interfere with BCG vaccination efficacy and in tuberculin test interpretation. Examining biogeographic trends in the distribution of members of the mycobacteria across a number of physicochemical and spatial gradients in soil and water environments across Ethiopia using oligotyping identified differential distributions of pathogenic and significant species. The tuberculosis complex was identified in more than 90% of water samples and taxonomic groups implicated in lower BCG vaccine efficiency were core in both soil and water *Mycobacterium* communities. A reservoir of *Mycobacterium bovis* was identified in water, with up to 7.3×10^2^ genome equivalents per ml. Elevation, temperature, habitat and vegetation type were important predictors of both soil and water *Mycobacterium* communities. These results represent the first step in understanding the potential risk of exposure to environmental mycobacteria that may undermine efforts to reduce disease incidence.

## Introduction

The *Mycobacterium* genus contains over one hundred and fifty recognised species which can be broadly grouped into fast-growing and slow-growing species or species complexes, based upon physiological, phenotypic and phylogenetic differences [1]. The Mycobacteria belonging to the slow-growing group are more commonly associated with host pathogenicity than those in the fast growing group [2] however, both groups contain pathogens causing pulmonary, disseminated and cutaneous diseases; particularly in immuno-compromised hosts [3–6]. The majority of the mycobacteria are found in the environment across a range of soil types and water distribution systems [7–13] which act as a reservoir for potential human and animal infection [14–17]. In addition to the potential for disease transmission, prior exposure to certain mycobacteria, including members of the *Mycobacterium avium* complex, have been implicated in the reduction of BCG vaccine efficacy against adult pulmonary TB in humans [18–22].

The geographical diversity, composition and abundance of environmental mycobacteria reservoirs are not well described, despite the need to address the risk of exposure in the context of disease, BCG vaccine efficacy, and tuberculin skin testing. The few studies that have identified environmental or geographical hotspots are based on relatively small numbers of samples and over limited geographic ranges; often by applying decontamination cultivation techniques considered to only reveal a fraction of the true diversity [10,13,23–25]. To overcome these limitations (particularly for complex environmental microbial communities), targeted amplicon-based pyrosequencing and post-sequencing analyses can provide accurate sequence information. However, the Mycobacteria are a closely related group of organisms showing little variation in the 16 rRNA gene [26] limiting the use of this target as a tool for uncovering diversity of this group. Oligotyping is more specific in targeting regions of conserved entropy in a multiple sequence alignment in order to separate sequences of closely related organisms during down-stream analysis [27].

Based on this oligotyping approach, *Mycobacterium*-specific pyrosequencing data were analysed to identify biogeographical patterns at the sub genera and species complex level in soil and water samples collected across Ethiopia. Ethiopia encompasses highly diverse and extreme climatic and environmental conditions, with one of the highest prevalences of all forms of tuberculosis worldwide [28] with an incidence rate of 247 per 100,000 [29] and a high rate of extra pulmonary tuberculosis [30]. Ethiopia also has widespread sensitivity to mycobacteria in humans and animals which may be the result of exposure to environmental mycobacteria [28,31–33]. The aims of this study were (i) to quantify the biogeographical variation in slow and fast growing environmental mycobacterial communities, (ii) to evaluate the associations between geographical, climatic and edaphic measures and the observed mycobacterial diversity and composition and (iii) to identify potential biogeographical hotspots of known pathogenic or opportunistic mycobacteria pathogen groups.

## Results

### Processed sequences results

*Mycobacterium* genus and slow-growing mycobacteria pyrosequencing primers detected a minimum of 380 sequence counts per soil and water sample after quality control. Using the *Mycobacterium* specific primers, 57 oligotypes were identified in soil and 41 groups in water samples. The slow-growing mycobacteria specific assay identified an additional 14 species groups in soils and 16 species groups in water.

### Core mycobacterial communities and pathogenic groups in soil and water

**Fig 1:**
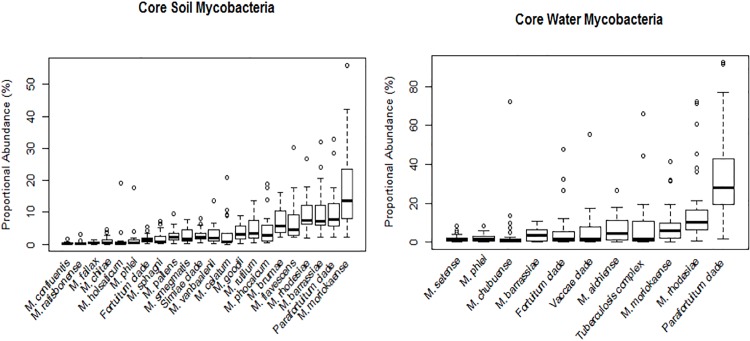
Mean proportional abundance of core mycobacteria community members. Proportional abundance of mycobacteria detected in > 90% of soil and water samples using the genus specific and slow growing specific primers respectively. Groups are ordered from left to right by increasing mean proportional abundance.

The core mycobacterial community members found in ≥90% of all soil and water samples were members of the Gordonae clade, Avium complex, and *Mycobacterium angelicum*. In addition, *Mycobacterium riyadhense* and Tuberculosis complex were also dominant in water samples (Fig 1).

44% and 38% of the *Mycobacterium* species groups detected in water and soils respectively are known to be pathogenic to humans. The most abundant *Mycobacteria* in both environments were *Mycobacterium moriokaense*, members of the Avium complex and the Parafortuitum clade. *Mycobacterium barrassiae* was as additional pathogen identified in soil. Tuberculosis complex members were present in all 42 water sample locations, and detected in two locations in soil where they contributed <0.2% of the total slow growing community in the latter substrate. QPCR demonstrated that the mean absolute numbers of mycobacteria genome equivalents were greater in the soil (1.60 ×10^8^ cells/gram; range: 9.47×10^5^ to 8.01×10^8^/gram) compared to in water samples (1.17 ×10^4^ cells/ml, range: 3.35×10^1^ to 2.68×10^5^/ml) (F[1,71] = 25.33, P<0.001). *M*. *bovis* specific qPCR returned counts in the range of 1.5–7.3×10^2^ gene copies per ml of water, and 2.9×10^3^ gene copies per gram in a single soil sample, indicative of a substantial reservoir source. The pathogens *M*. *tuberculosis* and *M*. *canettii* were not identified in any sample.

### Biogeographical variation in *mycobacterium* community composition and diversity

The diversity of soil mycobacterial communities at individual geographical locations (Simpsons alpha diversity index: mean 3.24, SD 0.24) was significantly greater than the diversity in the water communities, at both the genus level (mean 2.16, SD 0.64, Welsch’s two sample T-test: t(62) = 1.99, P<0.001) and for the slow growing mycobacteria (mean 1.94, SD 0.64, Welsch’s two sample t-test: t(72) = 1.99, P = 0.07).

The difference in mycobacteria species diversity between locations were significantly greater in soil (mean 101.13, SD 15.05) than across water sites (mean 58.41, SD 24.06, t(62) = 1.66, P<0.001), despite significant geographical heterogeneity in each substrate (soils: ANOVA: F(4,17) = 3.01, P<0.05; water: (F(8,33) = 2.0, P = 0.07). In contrast, the number of slow-growing oligotypes between substrates (F(1,72) = 0.63, P = 0.42) and between geographic regions, were not significantly different (P>0.1 for all comparisons).

### Environmental predictors of mycobacteria communities in water

**Fig 2:**
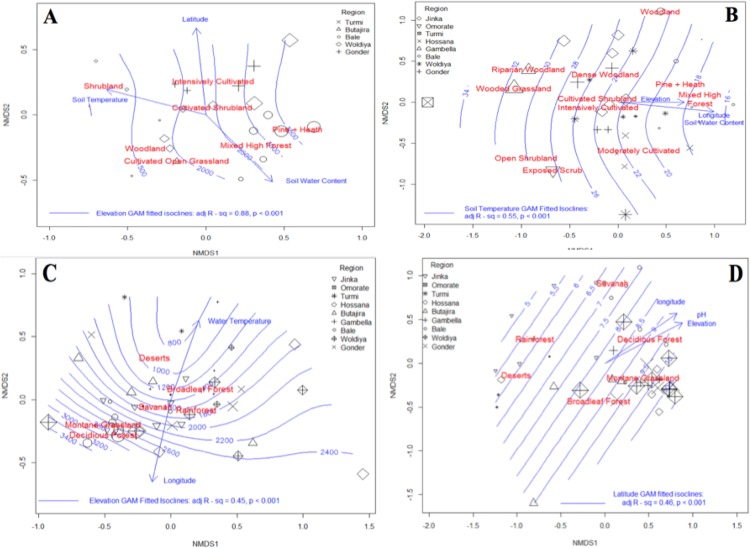
The effect of environmental variables on the presence of slow growing mycobacteria. NMDS ordination of slow growing mycobacterium water community’s highlighting the effect of each significant environmental variables upon the separation of the data points. GAM fitted isoclines represent predicted values for latitude and the size of each point represents the true value for this variable. A = mycobacteria communities in soil, B = slow growing mycobacteria in soil, C = mycobacteria in water, D = slow growing mycobacteria in water.

Elevation (R^2^ = 0.28, p<0.001), longitude (R^2^ = 0.27, p = 0.021) and water temperature (R^2^ = 0.37, p<0.001) were significant predictors of *Mycobacterium* community structure. Isoclines predicting elevation based on community dissimilarity were fitted to the ordination in the form of a GAM model (Adj R^2^ = 0.45, *P*<0.001) (Fig 2). Of the categorical variables measured, only habitat type was a significant predictor of mycobacteria community structure (R^2^ = 0.30, P<0.001).

**Fig 3:**
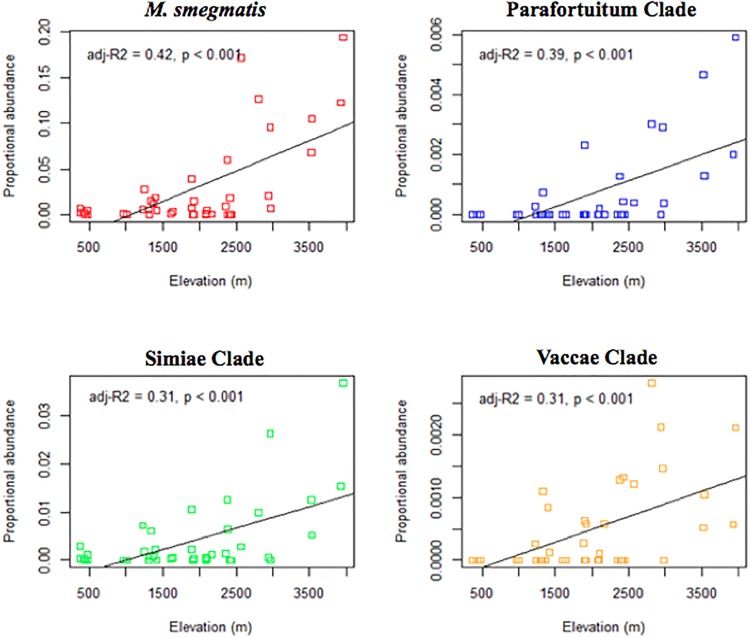
The relationship between elevation and mycobacteria abundance. Linear regression of the proportional abundance of mycobacteria in water against elevation.

Several known pathogen species and clades containing disease-associated members were found to significantly increase with increasing elevation (Fig 3). Beta diversity increased unimodally with altitude reaching a maximum at intermediate altitudes (2000-2500m) thereafter declining. Alpha diversity was not associated with any numerical variable.

### Environmental predictors of slow-growing mycobacteria communities in water

Slow growing mycobacteria community structure was significantly predicted by annual season temperature (R^2^ = 0.25, P = 0.005), latitude (R^2^ = 0.48, P< 0.001), pH (R^2^ = 0.21, P = 0.01) and elevation (R^2^ = 0.23, P = 0.026). Isoclines representing predicted values for elevation fitted to the NMDS ordination using a GAM model (R^2^ = 0.22, P<0.001) showed a linear effect across the second NMDS axis (Fig 2). The significant categorical predictors of slow growing mycobacteria diversity included vegetation classification (R^2^ = 0.54, P<0.001) and surrounding habitat type (R^2^ = 0.63, P<0.001) (Fig 2).

### Environmental predictors of mycobacteria communities in soil

Elevation (R^2^ = 0.81, P<0.001, GAM R^2^ = 0.80, P<0.001), annual season temperature (linear R^2^ = 0.80, P<0.001), latitude (linear ^2^ = 0.28, P = 0.05) and soil water content (R^2^ = 0.57, P< 0.001) were the numerical predictors of soil mycobacteria community composition when fitted to a NMDS ordination model. The categorical predictors included vegetation classification (linear R^2^ = 0.74, P<0.001), habitat type (R^2^ = 0.50, P = 0.002) and annual season precipitation (R^2^ = 0.28, P = 0.003) (Fig 2).

Alpha diversity was negatively correlated with soil water content (R^2^ = 0.227, P = 0.02). Unimodal relationships were observed between the number of mycobacterium genome equivalents per gram of soil (pseudo R^2^ = 0.317), community richness (pseudo R^2^ = 0.43), and abundance, which was highest at altitudes between 2000m and 3000m, and soil *Mycobacterium* diversity showed a linear increase with elevation (R^2^ = 0.50, P<0.001).

**Fig 4:**
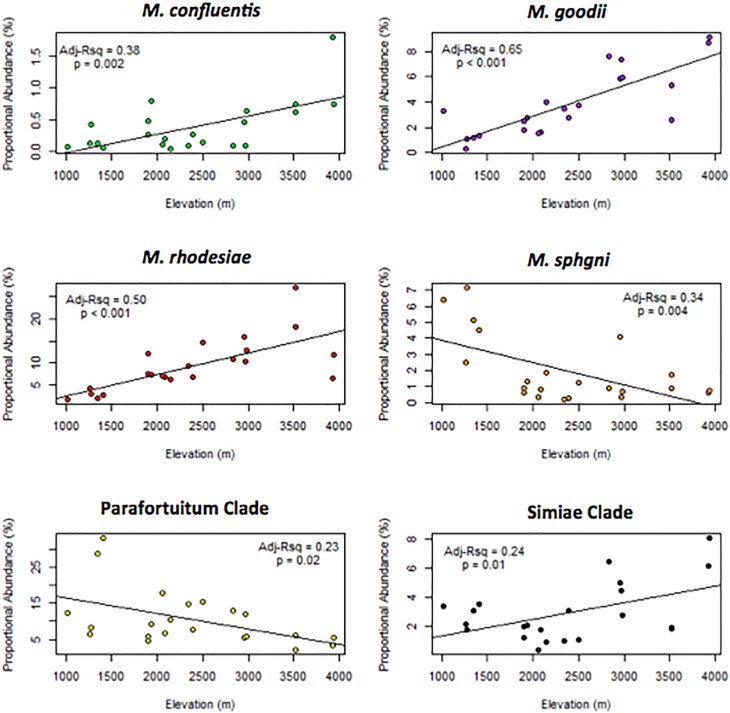
The effect of elevation on proportional abundance of soil mycobacteria. Linear regression of the proportional abundance mycobacteria in soil against elevation.

Several species known to cause disease and clades comprising disease-causing members were observed to significantly change in proportional abundance with changes in elevation: *M*. *confluentis* and *M*. *goodie* increased in abundance and *M*. *sphagni* decreased with increasing elevation (Fig 4).

### Environmental predictors of slow growing mycobacteria communities in soil

Similar to the genus level analysis, elevation (linear R^2^ = 0.30, P = 0.003), soil temperature (linear R^2^ = 0.43, P = 0.002), longitude (R^2^ = 0.48, P<0.001), soil water content (R^2^ = 0.41, P<0.001), vegetation classification (R^2^ = 0.63, P<0.001) and habitat type (R^2^ = 0.45, P<0.001) were significant predictors of the slow-growing mycobacterium community in soils.

**Fig 5:**
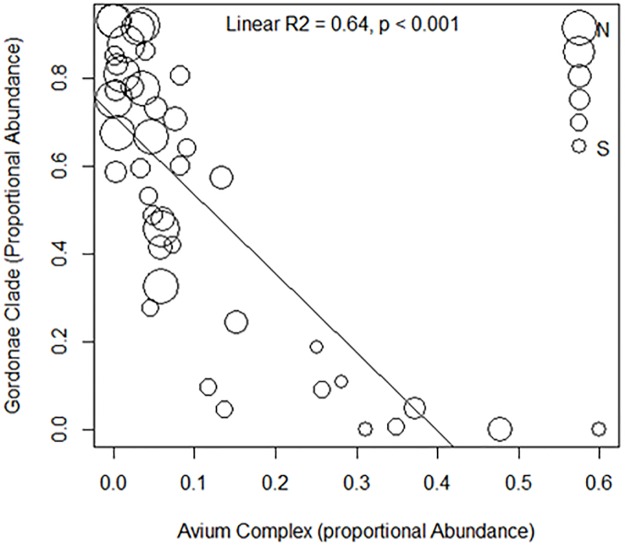
The proportional abundance of gordonae clade and avium complex in soil. Linear regression of the proportional abundance of the Avium complex against Gordonae clade in soil.

Alpha diversity increased with elevation (R^2^ = 0.34, P<0.001) and with longitude (R^2^ = 0.30, P = 0.01), and was correlated with the proportional abundance of Gordonae clade and the Avium complex (Avium complex: Pearson’s r = -0.53, P<0.001; Gordonae Complex: r = 0.52, P = 0.002). Areas with high proportional abundance of the Gordonae complex in the western locations in Ethiopia exhibited low proportional abundances of Avium complex members, and vice versa (Fig 5).

## Discussion

In this study a combination of oligotyping and qPCR were applied to soil and water ecosystem samples to understand the biogeographical structure and diversity of *Mycobacterium* communities measured across Ethiopian biomes. Fifty seven unique oligotypes were identified in water and seventy one identified in soils from combining results of the *Mycobacterium* genus and slow-growing specific assays. Particular significance was attributed to the slow growing groups, as many are known opportunistic pathogens of humans and animals. A high diversity and widespread distribution of mycobacteria was observed, particularly of the Gordonae clade, Avium complex and Tuberculosis complex in these environmental substrates. Particularly surprising, and of potential concern to the public and veterinary health communities, was the presence and relatively high abundance of Tuberculosis complex members identified in 100% of the water sample locations, comprising human and cattle drinking water sources. *M*. *bovis* was also detected in 2 village soil samples. *M*. *bovis* is the causative agent of tuberculosis in both cattle and humans, and in Ethiopia high prevalences have been recorded [34,35]. The presence of *M*. *bovis* in these sources suggests that water may be a reservoir for potential onward transmission. *M*. *bovis* is most likely to be present through contamination by visiting livestock which has previously been observed in Spain [36]. The same infected water sources were used by local communities for drinking, washing and bathing, highlighting a potential for cross-contamination to humans in addition to other animals. The relative contribution of water-borne verses air-borne transmission is not currently known, however water and sediment in the water is suspected as infection sources to animals elsewhere [37]. Furthermore, *M*. *bovis* residing in soil for over 12 months can colonise mice when eaten [38].

Members of the Mycobacterium Avium Complex (MAC) are also linked to clinical infections in humans and their livestock [39,40]. MAC was widely detected across the Ethiopian locations; though the distribution and proportional abundance varied substantially. Altitude was a significant predictor suggesting that human settlements situated at high latitudes (>5.733m) and at higher altitudes (>2951m) may potentially experiencing increased risk of exposure. MAC is commonly isolated from drinking water before and after water purification treatments [41,42] highlighting that water, in addition to soil, may be an epidemiologically significant source of potentially infectious material. Prior exposure to MAC is thought to “mask” or “block” human BCG vaccine efficacy against *M*. *tuberculosis* by interfering with the protective interferon-gamma responses following BCG vaccination [9,11,19]. Globally, the efficacy of BCG tends to decrease with latitude which has been suggested to be a consequence of a corresponding increase in abundance of environmental Mycobacteria. [19]. The current study is the first step in testing for the associations between BCG vaccine efficacy and EM compositions across Ethiopian biomes. In particular, the current data suggest that there is greater risk of exposure to MAC through water at higher altitudes. To further these developments it would be beneficial to characterise the communities of mycobacteria in the environment along a gradient of BCG vaccine efficiencies to determine which species may be producing the vaccine masking effects that have been hypothesised.

Variations in the genus and slow-growing taxa diversity were shown to be significantly associated with altitude, latitude and broad descriptions of the surrounding habitat type and vegetation. Particular species groups dominated water sources at higher elevations, namely *M*. *smegmatis*, the Parafortuitum clade, the Simae clade and Vaccae clade, whereas *M*. *rhodesiae* and *M*. *goodii* dominated high altitude soils. Soil mycobacteria exhibited a monotonic pattern in alpha diversity, oligotype richness and *Mycobacterium* copy numbers, showing a sharp decline above 4000m altitude indicative of peripheral viable growth conditions. Similar patterns were observed with elevation in the beta diversity within water sources: community variability was highest at intermediate altitudes and where water temperatures ranged between 17–23°C. Opposing trends between comparative water and soil substrates were observed, with the Parafortuitum clade in the water samples showing a significant increase in proportional abundance with elevation and the reverse pattern being observed in soil samples.

Analysis of the soil slow-growing *Mycobacterium* communities illustrated an inverse relationship between the the Gordonae and Avium complex proportional abundances, which was strongly associated with longitude. Higher Gordonae and lower Avium complex abundance was observed in western regions of Ethiopia, whilst the opposite pattern was observed in eastern Ethiopian sites.

This preliminary study demonstrates the importance of using high resolution techniques such as oligotyping to differentiate closely related taxa towards understanding the distribution of, and exposure risk to potentially pathogenic organisms in the environment. The genus *Mycobacterium* presents a particular challenge as it is characterised by very limited interspecies genetic variability [43] and robust phylogenies created using 16S rRNA genes can only be achieved for a small number of species groups [43]. The significance of environmental mycobacteria as reservoirs for onward transmission of pathogenic and opportunistic species to humans and livestock is poorly understood. Here heterogeneities in the potential environmental exposure risk were highlighted, particularly in regards to *M*. *bovis* and Avium complex members in water shared by human and animals across Ethiopia.

## Materials and methods

### Environmental sampling sites

Soil and water samples were collected from 42 villages in 21 districts (kebeles) in 9 administrative regions of Ethiopia. These regions were selected to represent the country’s climatic and environmental diversity. Mean annual temperatures ranged from <7.5°C—>27.5°C, varying particularly with elevation (range: 359-2983m). Mean annual rainfall ranged from < 200 mm to 2200 mm, being highest in western areas (1800–2000 mm) and lower in southern and eastern areas (<200 mm) (Ministry of Agriculture & Rural Development, Ethiopia). The sample sites were located between the latitudes 4°42’-12°46’N and longitudes 34°15–37°52’E.

### Elevation transects

**Fig 6:**
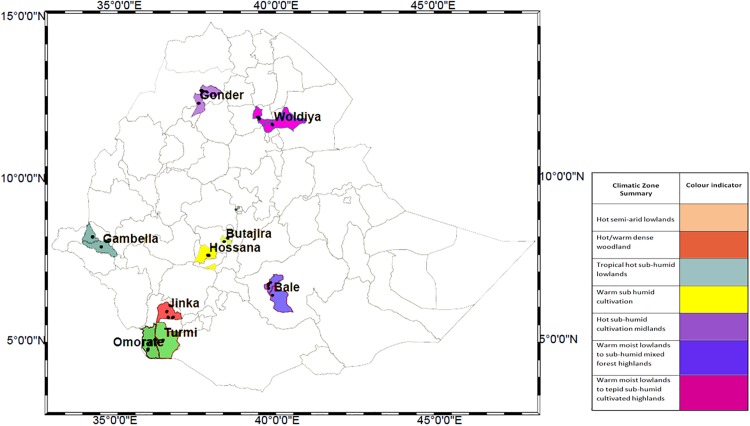
Map of sample collection locations. Map of Ethiopia to show the sampling locations, shaded areas represent climatic descriptions of the nine regions sampled.

Eight villages were sampled along each of two elevational transects, one in Woldiya and the other in the Bale region (Fig 6) in order to determine the environmental and climatic effects associated with changes in elevation within a relatively short geographical distance. The eight Woldiya villages were located within 61km over an elevation range of 1414m to 3396m, and the Bale villages were located within 54km over an elevation of 1224m to 3997m.

### Sample collection and environmental readings

In each village water was sampled from two sampling points including the predominant communal areas for drinking and bathing including rivers, small streams and sealed wells. As sample collection did not involve humans the AHRI-ALERT Ethical review committee deemed ethical approval unnecessary for this study. The University of Warwick approved the study. Informed verbal consent was obtained from all land owners prior to sample collection. Soil was imported to the University of Warwick using DEFRA licence number PHL 208/6138. This study did not involve endangered or protected species.

In each of the 42 villages, soil was sampled from five sites including two households, two household yards and one communal site such as a school or market where the top 3cm of soil and vegetation was removed prior to sample collection to reduce the effect of UV irradiation. At each of the five sites (<2 kilometres apart), soil was collected using a sterile trowel into a plastic bag from three areas (metres apart) and immediately pooled to make a composite sample. The plastic bag was shaken and three replicates of approximately 5g taken immediately and stored in bijou tubes. The composite samples were stored at -20°C. For water sample collection, two sites for each of the 42 villages were chosen comprising of one drinking source and one bathing source (often these were interchangeable). Homes did not have piped running water. Samples were collected from a variety of sources including groundwater, drinking pumps, springs, rivers and lakes. At each sampling point, 100ml of water was collected from the surface and was filtered using a 50ml sterile plastic syringe and the MicrofilV filtration device with 0.22μm mixed cellulose esters white gridded filters (Millipore, MA, USA). After collection the filters were removed from the plastic holder using sterile forceps and air-dried. The filters were then coiled and stored in 5ml bijou tubes in a cool box with ice packs (4°C) for transporting to the laboratory where the filters were stored at -20°C. All samples were collected during the dry season (Jan–March 2010).

Biogeographic measures were recorded for each site using an eTrex Legend H Outdoor GPS (Garmin Ltd., UK). Readings for soil moisture were taken at each soil sampling site using a soil moisture sensor (SM200 Delta-T Devices Ltd., UK) and moisture meter (HH2 Moisture Meter, Delta-T Devices Ltd., UK) where the output was volumetric water content expressed as a percentage. Substrate temperature and pH readings were taken using a Field Scout SoilStik (Spectrum technologies, Inc., IL, USA). The three readings were averaged per sample type (soil and water) and this average used for statistical analysis. The variables tested by multivariate analysis included temperature, elevation, moisture, pH, latitude and longitude.

### DNA extraction

Metagenomic DNA was extracted from 0.5–0.6 g of soil using the FastDNA Spin Kit for Soil (MP Biomedicals, OH, USA) according to the manufacturer’s instructions. The soil community DNA was extracted from the five sampling points and pooled to represent the soil community DNA for the village for pyrosequencing. Metagenomic DNA was extracted from the water polycarbonate filters using the PowerWater DNA Isolation Kit (MoBio Laboratories, Inc., CA, USA) according to manufacturer’s instructions. Samples were normalised using readings from the NanoDrop 1000 spectrophotometer (NanoDrop products, Wilmington, DE, USA) so that equal amounts of nucleic acid were represented from each of the sites for one village.

### Quantitative PCR assays

A real-time quantitative PCR (qPCR) assay was performed to determine *Mycobacterium* genus abundance by targeting the internal transcribed spacer and partial 23S gene (23, 31). The total volume was per reaction 25μl, comprising of 12.5μl of TaqMan Environmental master mix 2.0, 0.4 μM of each primer and probe, 0.4 μM bovine serum albumin (BSA), and 1 μl of the total community DNA. Thermocycle conditions were 2 min at 50°C, followed by 10 min denaturation at 95°C, then by 40 cycles of 15 s at 62°C and 95°C for 1 min. *Mycobacterium tuberculosis* DNA dilutions were prepared to generate a standard curve which ranged from 5.8 x 10^5^ gene copies μl^-1^ to 5.8 x10^1^ gene copies μl^-1^.

A quantitative PCR assay was employed to determine the abundance of *M*. *bovis* in environmental samples. The previously designed qPCR assay targets the RD4 scar region specific for *M*. *bovis* (33). All procedures and reagents were as previously published (Sweeney et al, 2007).

A multiplex qPCR assay was employed to quantify the abundance of the *M*. *tuberculosis* complex and abundance of *M*. *tuberculosis*/*M*. *canettii* (32). For each reaction, the total volume was 30μl, comprising of 15μl of TaqMan Environmental master mix 2.0 (Applied Biosystems Inc., CA, USA), 0.6μl of each primer and probe, 1μM BSA, 2μl of Mycobacterium smegmatis DNA (1.22 x10^3^ cell copies per ml) and 1μl of the total community DNA. Primer and probes are referred to as MTC_IAC Fw, MTC_IAC_Rv, MTC probe, IAC probe, wbbl1_Fw, wbbl1_Rv and wbbl1 probe in the previously published paper (32). Thermocycle conditions were 2 min at 50°C, followed by 10 min at 95°C, then by 40 cycles of 15 s at 95°C and 58C for 1 min.

All qPCR reactions were carried out on the ABI 7500 Fast Real-Time PCR System (Applied Biosystems Inc., CA, USA).

### Primers targeting *mycobacterium* 16S rRNA gene for pyrosequencing

The Mycobacterium genus was targeted for amplicon pyrosequencing using published primers (JSY16S) amplifying the variable regions 2–4 of the 16S rRNA gene (34). In a separate reaction a second set of primers specific to a group of slow-growing mycobacteria (APTK16S) was employed as it was shown that the Mycobacterium genus primers (JSY16S) were biased toward the detection of fast-growing mycobacteria. This targets the variable regions 1–3, where it specifically targets the long helix 18 insertion present in the majority of slow-growing mycobacteria (Leclerc *et al*, 2003).

### Bacterial tag-encoded FLX amplicon pyrosequencing

Sequence libraries were constructed using the HotStarTaq Plus Master Mix Kit (Qiagen Ltd., UK) and a one-step PCR with 30 cycles. Bacterial tag encoded amplicon pyrosequencing was carried at the Research and Testing Laboratory (Lubbock, TX) using a Roche 454 FLX instrument with titanium reagents.

### Pyrosequencing quality control

The data was quality controlled using QIIME procedures. Sequences were removed if the length was <400bp for the *Mycobacterium* genus (JSY16S) and <420bp slow-growing mycobacteria (APTK16S) datasets respectively. Sequences were retained if they matched the proximal primer with one mismatch or less and if the sequence had an average quality score of ≥25. Sequences were denoised using the Denoiser in QIIME and chimeric sequences were identified and removed using ChimeriaSlayer. In order to determine the minimum number of sequences needed per sample for sample inclusion, initial rarefaction curves were generated using OTUs at clustered at 97% similarity using UCLUST (Edgar, 2010). Across all datasets, samples were excluded if they had less than 380 sequences after quality control procedures. Samples with > 380 sequences reached an asymptote and were considered comparable.

### Oligotype generation

Sequences were aligned using PyNAST (Caporaso *et al*, 2010) and entropy components were calculated using the Shannon’s entropy script in the oligotyping suite. Entropy components were chosen in a supervised fashion where *H* exceeded 0.6 and the position in the alignment was overlapped by all sequences.

### BLAST assigned taxonomy

Representative sequences were chosen from each oligotype based upon the most frequent sequence and queried using a local Blast algorithm against a customised database containing all mycobacterium sequences in the SILVA database (1019 sequences). Genus level names were altered to reflect the species complex, group or clade where applicable. An acceptable sequence match was retained if the E value was <0.001 and had >90% identity to the candidate sequence.

### Statistical analysis

For all pyroseqencing datasets non-metric multidimensional scaling (NMDS) plots were used to determine significant predictors of mycobacteria community structure using oligotype frequency matrices in R. Environmental variables were fitted to the plot in the form of a least squares regression model to determine the best predictors of mycobacteria community structure using the envfit routines in R. Specific environmental variables showing significant linear trends across the NMDS plots were highlighted by adding isoclines of predicted values using a general additive model approach using the ordisurf routines in R. Plotted points were scaled in size to represent the true values of the GAM fitted environmental gradient. Shannon’s diversity index *H’* was calculated on rarefied oligotype matrices using vegan in R. Pearson’s correlation coefficient was calculated to statistically test relationships between individual oligotype prevalence and bioclimatic and spatial variables. Resulting probability values were Bonferroni corrected. Beta diversity was calculated after bining the samples on the basis of the predictor variable and calculating the sum of the proportional abundance of each oligotype minus the mean proportional abundance of that oligotype within each group. Beta diversity was calculated by subtracting the proportional abundance of each oligotype within an environmental bin from the mean proportional abundance of that oligotype across all samples within each environmental bin
